# Versatile Polypropylene Copolymers from a Pilot-Scale Spheripol II Process

**DOI:** 10.3390/polym12040751

**Published:** 2020-03-30

**Authors:** Xiong Wang, Renwei Xu, Wenqian Kang, Jie Fan, Xiaoyu Han, Yunbo Xu

**Affiliations:** Lanzhou Petrochemical Research Center, Petrochemical Research Institute, PetroChina, Lanzhou 730060, China; xurenwei@petrochina.com.cn (R.X.); kangwenqian@petrochina.com.cn (W.K.); fanjie3@petrochina.com.cn (J.F.); hanxiaoyu@petrochina.com.cn (X.H.); xuyunbo@petrochina.com.cn (Y.X.)

**Keywords:** polypropylene polymerization, Spheripol process, random PP (RPP), impact PP, impact PP with high clarity

## Abstract

Polypropylene (PP) is one of the most widely used polymers. In this paper, three types of PPs including random PP, impact PP, and impact PP with high clarity, were prepared through a 75 kg/h pilot-scale Spheripol II process. The three produced PPs were produced by the selection or combination the two loops and gas phase reactor and controlling the comonomer and hydrogen concentrations. The three prepared PPs then were pelleted with the clarified nucleating agent NX 8000 and tested for mechanical, thermal, and optical properties. Their molecular structures and rubber phase size were also investigated by GPC, ^13^C NMR, temperature rising elution fractionation (TREF), XRD, SEM analysis, etc. The results showed that the random PP (PP-1) and the impact PP with high clarity (PP-3) obtained excellent optical transparency with a haze of 12.5% and 13.5% due to their small rubber phase size (roughly ≤ 100 nm), while the impact PP (PP-2) obtained bad transparency with a haze of 98.8% due to the large rubber phase size (about 1 μm) caused by the poor thermal compatibility with the PP matrix. The rubber phase content and ethylene/propylene sequence distributions of the three PPs varied much and resulted in different impact strengths and stiffness properties. PP-2 had a high impact strength of 14.5 kJ/m^2^ due to the rubber phase generated in the gas phase reactor. Except for the optical transparency, PP-3 gained stiffness and toughness, with 914 MPa of flexural modulus and 25.1 kJ/m^2^ of impact strength due to the unique molecular structure of its rubber phase.

## 1. Introduction

Since the discovery of the Ziegler–Natta (Z-N) catalyst in the 1950s, the production of polyolefins with various chain microstructures and properties has continuously grown with the rapid development of catalyst technology combined with polymerization innovation [1,2,3,4,5,6,7,8,9,10]. Polypropylene (PP) is undoubtedly one of most used and robust material fields in the production and consumption market globally [11,12,13,14,15]. Its properties vary from plastic to elastomer, and it is used in a wide range of applications, from packaging to household electric appliances, medical, lightweight engineering plastics for automobiles, construction, equipment, and facilities industries. The global production of PP was about 56 million tons in 2016 and is estimated to reach 80 million tons by 2022 [16]. 

The first major breakthrough in the Z-N catalysts in 1968 was promoted by Montedison (now LyondellBasell) and Mitsui with the discovery of the milled MgCl_2_ support for ethylene polymerization [17,18,19]. This technology was adopted by the extra addition of internal and external electron donors to improve the isotacticity of PP without catalyst activity in the PP industry in the 1970s, which led to the third-generation of PP catalysts, eliminating the need for catalyst residue removal, but the atactic content was still too high.

The second breakthrough appeared with “the reactor granule technology” (RGT) in the fourth-generation catalysts in the 1980s. When propylene polymerization occurred in the RGT catalyst, the catalyst grew into a polymer particle with active sites within it, so both the catalyst and the polymer particle could act as the reactor during the polymerization.

The heterogeneous catalysis was mainly based on the active MgCl_2_ in a spherical granule or spherical form with MgCl_2_ or Mg(OC_2_H_5_)_2_ as a starting material [20,21,22,23]. Those granule or spherical MgCl_2_-based PP catalysts typically had a higher and longer activity and were capable of tuning the molecular distribution by the selection of internal electron donors, allowing morphology replication of the support particle in the process of polymerization; therefore, substantial process simplification was made possible. Then, the innovation in Z-N PP catalyst in recent decades was mainly focused on alternative electron donors (including internal and external donors) to develop PP with specific molecular microstructures or even higher activity [24,25,26,27,28,29]. 

Along with the development of PP catalyst technology, a series of PP polymerization processes was developed and commercialized successfully. Owing to the RGT catalysts, the revolutionary development of PP-based production processes also took place, such as Spheripol, Novelen, Spherizone, Unipol, Catalloy, etc. [16,30,31,32], which made it possible to generate multiphase alloys and blends directly in multiple reactors, producing high-performance materials not available with conventional technologies. 

In this work, a spherical MgCl_2_-based Z-N catalyst was used in 75 kg/h pilot-scale Spheripol II equipment. Generally, isotactic polypropylene (iPP) and random copolymer polypropylene (RPP) can be produced in the first two loops, while impact PP can be produced by the first two loops and the third gas phase reactor. By tuning the polymerization conditions, three types of PP, including an ethylene/propylene/1-butene (EPB) random copolymer, an impact ethylene/propylene (E/P) copolymer, and a transparent and impact polypropylene, are generated in the Spheripol II process. The three types of PP are characterized by GPC, ^13^C NMR, temperature rising elution fractionation (TREF), XRD, SEM analysis, etc. The results show that the three PPs possess different molecular chain microstructures, which result in various mechanical, thermal, and optical properties.

## 2. Materials and Method

### 2.1. Materials 

Ethylene, propylene, 1-butene, and nitrogen gas (polymerization grade, ≥ 99.99%) were provided by Lanzhou Petrochemical Company, PetroChina (Lanzhou, China). Three types of polypropylene (PP) copolymers were prepared through different process conditions via 75 kg/h pilot-scale Spheripol II process equipment at Lanzhou Petrochemical Research Center, Petrochemical Research Center, PetroChina. A conventional Z-N catalyst (provided by Lanzhou Petrochemical Company, PetroChina) was used in the polymerization. Antioxidant 1010, antioxidant 168, calcium stearate, and nucleating agent Millad NX8000 were donated by Lanzhou Petrochemical Company as PP additives. 

### 2.2. Polymerization Process and Prepared Copolymers

**Figure 1:**
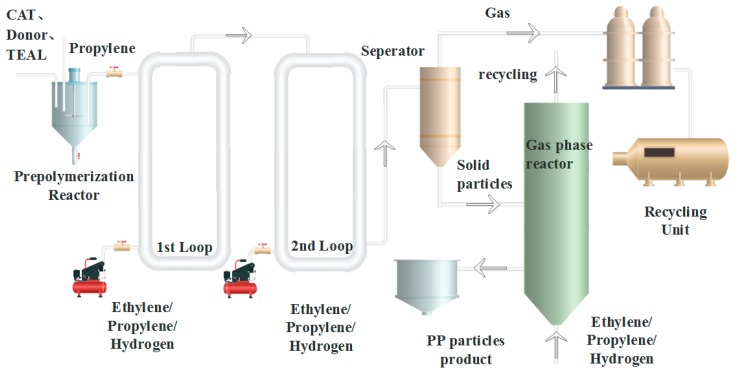
The schematic process of Spheripol II. CAT: Z-N catalyst, TEAL: triethylaluminum, Donor: cyclohexyl methyl dimethoxysilane.

Propylene and ethylene polymerization were conducted in a 75 kg/h pilot-scale Spheripol II process. The schematic PP process of Spheripol II is shown in Figure 1. As seen in Figure 1, the Spheripol II process consisted of two loops and a gas phase stirring reactor. Generally, propylene homopolymerization or random polymerization with ethylene or α-olefin could be conducted in the two loops, while block copolymerization was only conducted in the gas phase reactor.

The catalyst used was TiCl_4_ supported on MgCl_2_ with triethylaluminum cocatalyst, diisobutyl phthalate as the internal electron donor, and cyclohexyl methyl dimethoxysilane as the external donor. Three types of PP were prepared according to different polymerization processes and conditions, and they are listed in Table 1. PP-1 was prepared through random copolymerization of propylene with ethylene and 1-butene, and the gas phase reactor was not used. PP-2 was prepared through random copolymerization of propylene and ethylene in the two loops, combined with block copolymerization of propylene and ethylene in the gas phase reactor. PP-3 was prepared through propylene homopolymerization in the first two reactors combined with block polymerization of propylene and ethylene with a certain concentration of hydrogen. Then, the prepared PP particles were mixed with a certain amount of additives in a high-speed mixer and extruded and made into pellets using a twin-screw extruder (ZSE-34, LEISTRTIZE, Wiesbaden, Germany).

### 2.3. Characterization

#### 2.3.1. Mechanical Test

The tensile and flexural properties were tested on an Instron 5566 universal testing machine (Instron, Norwood, MA, USA) at room temperature (23 °C) according to GB/T 1040.1-2006 and GB/T 9341-2008, respectively. A CEAST 7028 (CEAST, Turin, Italy) was used to test the melting index according to GBT 3682, and the heat distortion temperature (HDT) was tested on an XRW-300UA (Zhonghangshidai, Beijing, China) according to GB/T 1634.1-2004. The notched Izod impact was carried out on a 92T Pendulum impact tester (TINIUS OLSEN, Philadelphia, PA, USA) at 23 °C according to GB/T 1843-2008. All test specimens were kept at 23 °C for at least 24 h before the test, and the average value was taken from at least 5 tests.

#### 2.3.2. IR, DSC, and XRD Analysis

IR analysis was performed on a NEXUS 670 FT-IR (Nicolet, Glendale, WI, USA). Film samples of about 10–20 μm were prepared for testing at 180 °C and under 10 MPa pressure. Differential scanning calorimetry (DSC) analysis was carried out on a DSC 214 Polyma instrument (NETZSCH, Selb, Germany). Seven to ten milligrams of sample were firstly heated from room temperature to 200 °C under a nitrogen atmosphere at a heating rate of 20 °C/min, then cooled to 30 °C at a cooling rate of 20 °C/min to eliminate the heat history of the sample. The melting and crystallization curves were obtained, respectively, when heating the samples to 200 °C and cooling the samples to 30 °C at the same heating and cooling rate once again. Wide-angle X-ray diffraction (WXRD) was tested on a D8 ADVANCE diffractometer (Bruker, Karlsruher, Germany). A 1 mm thick sheet of the samples was used and scanned at 40 °C and 4°/min under Cu-Kα irradiation (λ = 0.154 nm).

#### 2.3.3. GPC and ^13^CNMR Analysis

Gel permeation chromatography (GPC) was carried out on a GPC-IR instrument (Polymer Char, Valencia, Spain) at 135 °C using 1,2,4-trichlorobenzene (TCB) as the solvent with a sampling concentration of 3 mg/mL and a sampling rate of 1.0 mL/min. The ^13^C NMR spectra of samples were recorded on a Bruker 500 MHz NMR spectrometer (Breika, MA, USA) at 120 °C using o-C_6_H_4_Cl_2_/o-C_6_D_4_Cl_2_ (50% *v/v*) as the solvent. The ^13^C NMR spectra were obtained with a 74° flip angle, an acquisition time of 1.5 s, and a delay of 4.0 s.

#### 2.3.4. Solubility in Xylene and TREF Analysis

Xylene soluble analysis was performed according to GB/T 24282-2009. A 2.0 g sample was dissolved in 100 mL xylene at 130 °C, then the solution was cooled to 25 °C and sieved to remove the solid and obtain the soluble xylene by rotating evaporation. Temperature rising elution fractionation (TREF) was analyzed in a Model 200+ instrument (Polymer Char, Valencia, Spain). Standard conditions were used with 40 mg in 20 mL of 1,2,4-trichlorobenzene (TCB), a crystallization rate of 0.5 °C/min, and an elution rate of 1 °C/min.

#### 2.3.5. Crystallization and Rubber Phase Morphology Observation

Isothermal crystallization was observed through a polarized optical microscope (DM2500P, Leica, Weztlar, Germany) at 140 °C for 5 min. Scanning electron microscopy (SEM) was performed on an ULTRA plus field-emission electron microscope (FESEM, Zeiss, Oberkochen, Germany). The specimens were prepared by the cryogenic fracture of the injection molded bars under liquid nitrogen (77 K) for at least 10 min and then etched in xylene at room temperature for 24 h to remove the rubber phase. Then, the fracture surface was coated with a thin layer of gold-palladium in a vacuum before the observation of the surface morphology.

## 3. Results and Discussion

### 3.1. Mechanical and Optical Results 

Generally, a series of additives including antioxidants, nucleating agents, acid absorbents, etc., was mixed with the PP powder to improve the overall performance of the final product. In this work, nucleating agent NX8000 was incorporated into the three PPs to evaluate their mechanical properties, as well as optical properties. As seen from Table 2, the mechanical properties of PP-1 could be improved dramatically when the amount of NX8000 was increased from 0 ppm to 3000 ppm, with the impact strength increased from 3.5 kJ/m^2^ to 6.5 kJ/m^2^ and a flexural modulus from 891 MPa to 1162 MPa. Moreover, the nonitol-based Millad NX 8000 exhibited an excellent clarifying effect by decreasing the size of spherulites and improved the transparency with the haze decreased from 45.1% to 12.5%.

The three types of PP prepared from the pilot-scale Spheripol process equipment were also tested to evaluate their overall mechanical and optical performance using the same amount of NX 8000. As shown from Table 2, the melting index of three types of PP was well-controlled between 8 and 11 g/10 min, which endowed them a similar processability. PP-1 prepared from random copolymerization had the best stiffness with a flexural modulus of 1162 MPa and a tensile yield stress of 28.3 MPa; however, its toughness was the lowest in terms of the impact strength of 6.5 kJ/m^2^ without using the ethylene/propylene copolymerization in the third gas reactor. In contrast, when ethylene/propylene gas copolymerization was adopted for PP-2 and PP-3, they acquired excellent impact strength with that of PP-2 of 19.6 kJ/m^2^ and of PP-3 of 25.1 kJ/m^2^, with a compensation of stiffness for toughness. The heat distortion temperature (HDT) of PP-1 was 76 °C due to its high stiffness. Although the flexural modulus of PP-3 (914 MPa) was much higher than PP-2 (739 MPa), the HDT of PP-3 (62 °C) was lower than that of PP-2 (69 °C), which may be attributed to their different rubber phase sizes.

**Figure 2:**
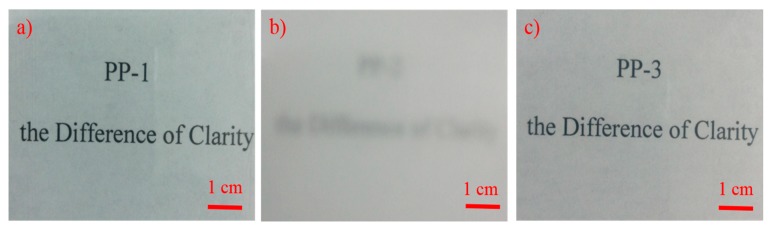
Digital photos comparing the transparency of various polymers (1 mm thick sheet). (**a**) PP-1; (**b**) PP-2; (**c**)PP-3. Three samples were determined with 3000 ppm NX 8000 as the nucleating agent.

Despite the similar polymerization process of the combination of two loops and a gas reactor, the difference in the rubber phase size also led to the change of optical results: the haze of PP-3 (13.5%) was lower than PP-2 (98.8%) due to the smaller size of the rubber phase dispersed in PP-3 (we will discuss the rubber phase size later in detail). For transparent copolymers, the rubber phase and the spherulite sizes were normally below the half wavelength of the visible light to avoid strong light scattering. PP-1 had the best optical properties due to a lack of a rubber phase generated in the gas reactor. From Figure 2, we can observe that PP-1 and PP-3 had excellent transparency with a legible view of the underlying paper through a 1 mm thick sheet, while being illegible for PP-2, mainly due to its large rubber phase size. Typically, PP-3 not only offered a great stiffness-toughness balance, but excellent optical transparency, compared to PP-2 prepared through a similar process.

### 3.2. Molecular Structure Analysis

#### 3.2.1. Solubility in Xylene and IR Analysis 

The three PP materials prepared from different polymerization processes have different chemical compositions and xylene soluble fractions at 25 °C. The ethylene and 1-butene monomer content could be determined from IR spectrum according to [33,34]. The signal ranging from 4482 cm^−1^ to 3950 cm^−1^ of the PP matrix could be used as a calibration reference, when ethylene content was less than 8%; the absorption peak signal from 758 cm^−1^ to 679 cm^−1^ could be adopted for the determination of the ethylene content; and the signal from 777 cm^−1^ to 679 cm^−1^ could be used when the ethylene content was above 8%. The peak around 769 cm^−1^ could be used to characterize the 1-butene content. Therefore, the value of the peak area of ethylene or 1-butene from 4482 cm^−1^ to 3950 cm^−1^ of the PP matrix could be used to calculate the ethylene or 1-butene content by standard curves. The monomer contents and xylene soluble fraction results are listed in Table 3.

The ethylene and 1-butene content in the ethylene/propylene/1-butene (E/P/B) random copolymer PP-1 was about 3.0% and 1.1%, respectively. When the third gas phase reactor was adopted to generate the E/P rubber phase, PP-2 obtained a higher ethylene content of 8.0%. Meanwhile the ethylene content of PP-3 was only about 4.9% due to the homopolymerization of propylene in the two loops. The ethylene or 1-butene content of the three copolymers (PP-1/PP-2/PP-3) could be easily controlled by changing the amount of monomers in the three reactors.

The xylene soluble analysis was also conducted to characterize the atactic PP or the amorphous rubber phase, especially the E/P rubber phase generated in the gas phase reactor. As illustrated in Table 3, the xylene soluble fraction at 25 °C of random copolymer PP-1 was 4.8%; when E/P copolymerization was conducted in the gas phase reactor, the xylene soluble fraction of PP-2 and PP-3 increased to 15.2% and 12.2%, which indicated that PP-2 had the highest E/P rubber content. We could also see a similar trend of ethylene content in the three PPs from the IR analysis of the soluble fraction and non-soluble fraction in xylene, with ethylene content in the soluble fraction of PP-2 of about 35% and PP-3 of about 25%.

#### 3.2.2. GPC Analysis

**Figure 3:**
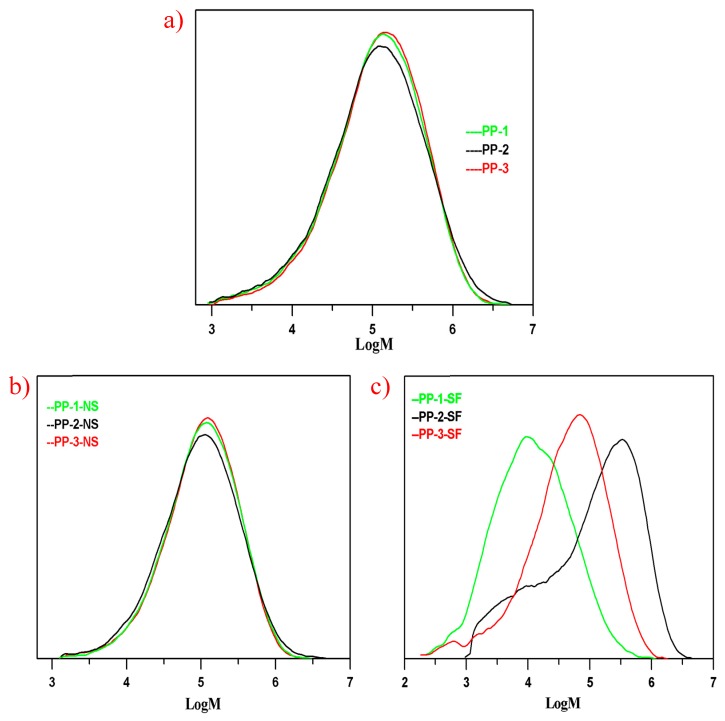
Molecular weight distribution curves of (**a**) PP samples, (**b**) non-soluble, and (**c**) soluble fractions from xylene at 25 °C.

From the discussion above, PP-1 was typically a random transparent copolymer, PP-2 an impact copolymer, and PP-3 an impact copolymer with high transparency. The rubber phase or the xylene soluble fraction and the rubber phase size dispersed in the PP matrix played a significant role in the mechanical and optical properties of the three PPs. In order to investigate how the rubber phase size dispersed in the PP matrix was influenced by the molecular structure of the copolymers, the molecular weights and molecular weight distributions of the three PPs and their xylene soluble fractions were determined by GPC analysis, and the GPC results are shown in Table 4 and Figure 3. 

Due to the similar melting index, the average molecular weight (Mw) of the three PPs did not vary much, with the Mw of PP-1, PP-2, and PP-3 being 221,700 g/mol, 245,300 g/mol, and 228,100 g/mol, respectively. As seen in Figure 3, there were more small molecules with Mw below 10^4.5^ (Mw roughly about 32,000 g/mol) than in PP-1 and PP-2, which could be mainly attributed to random copolymerization conducted in the two loops. The molecular weight distribution of PP-2 (PDI = 6.73) was broader than PP-1 (PDI = 5.79) and PP-3 (PDI = 5.33), which could be well explained by the small molecule chains produced in the loops and the large molecules generated in the third gas phase reactor.

The molecular weight distribution curves of the xylene soluble fractions in the three PPs would help further to illustrate the molecular chain structures produced in different reactors. The soluble fraction in xylene at 25 °C of PP-1 as mainly composed of small molecules with an average Mw of 29,700 g/mol; by the same token, the random E/P copolymer produced in the two loops also composed one part of the xylene soluble fraction in PP-2. As seen in Figure 3c, except for the small molecules in PP-2, the other part of xylene soluble fraction in PP-3 consisted mainly of the large E/P rubber chains. In contrast, the soluble fraction was mainly generated in the gas phase reactor, the average molecular weight of the soluble fraction in PP-3 being 95,800 g/mol, lower than that of PP-2 of 228,100 g/mol. Therefore, the molecular weight distribution of xylene soluble fraction in PP-1 (PDI = 6.65) was the narrowest due to the relatively small molecule chains, and the PDI of SF in PP-2 (15.45) was the broadest because of both the random small molecules and the large E/P rubber molecules.

On the other hand, the molecular chain structure in the soluble fractions seemed to play a significant role in forming the rubber phase size when melt blending of semi-crystalline polymers and amorphous rubber chains. The amorphous polymers with small molecule chains would have better thermodynamic compatibility with the semi-crystalline polymer chains due to less chain entanglement. Consequently, the xylene soluble fractions in PP-1 had the best thermodynamic compatibility with its crystalline matrix, while PP-3 had the biggest rubber size due to poor thermodynamic compatibility. The different transparencies of the three PPs could also corroborate the difference of rubber size in different PPs, and the rubber phases of the three PPs were shown by the SEM microscope images.

#### 3.2.3. ^13^CNMR Analysis

**Figure 4:**
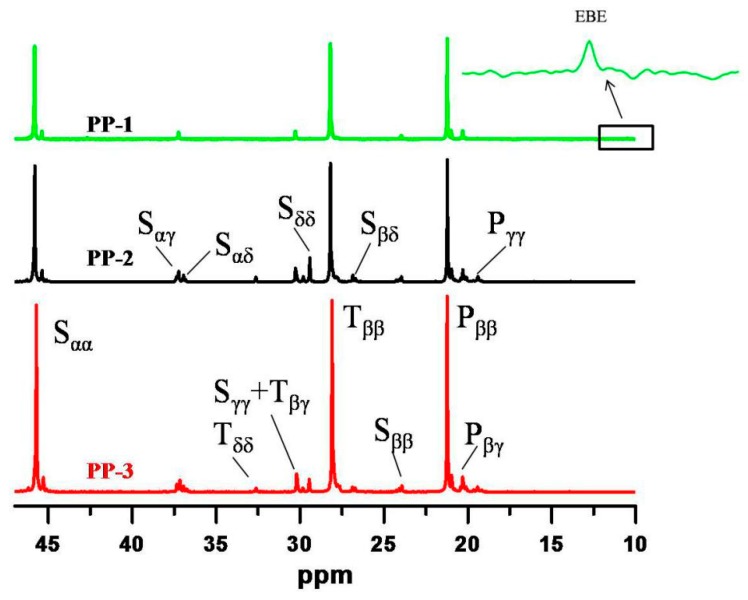
^13^C NMR spectra of the obtained samples PP-1, PP-2, and PP-3 measured at 120 °C using o-C_6_H_4_Cl_2_/o-C_6_D_4_Cl_2_ (50% *v/v*) as the solvent.

The comonomer sequence distribution of the three PPs was characterized by ^13^C NMR at 120 °C using o-C_6_H_4_Cl_2_/o-C_6_D_4_Cl_2_ (50% *v/v*) as the solvent. As seen in Figure 4, a tiny resonance peak around 10.5 ppm was observed for the random copolymer PP-1, indicating that a trace of alternating EBE (ethylene-1-butene-ethylene) sequences was present. For simplicity, 1-butene was negligible for microstructure analysis of PP-1, and only ethylene/propylene sequences were calculated for PP-1. The triad and diad sequence distributions of ethylene/propylene in the three PPs were calculated according to the method proposed by Carman and Randall et al. [35,36], and the results are presented in Table 5. From the ^13^C NMR results, the EEP/PEE sequence molar fractions varied from 0.37% to 9.91%, which might be attributed to the different xylene soluble fractions in the three PPs.

### 3.3. DSC and XRD Analysis

**Figure 5:**
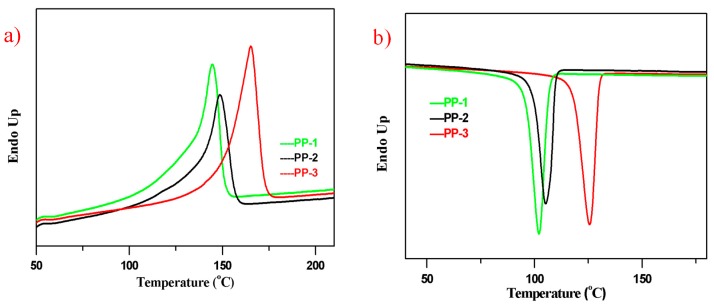
DSC curves for thee PP pellets at 20 °C/min. (**a**) Melting curves; (**b**) cooling curves.

Differential scanning calorimetry (DSC) results displayed (Figure 5) that the three PPs had different melting and crystallization temperatures, indicating different molecular structures. PP-3 had a melting peak around 165 °C, which was typically attributed to the highly isotactic polypropylene chains. The melting peak around 149 °C of PP-2 belonged to the E/P random copolymer chains, and the insertion of ethylene monomer reduced the stereoregularity of the isotactic PP chains, thus decreasing the melting point. Similarly, the E/P/B random copolymer PP-1 had a melting peak around 145 °C.

The crystallization peaks of PP-1, PP-2, and PP-3 were about 102 °C, 105 °C, and 125 °C, respectively.

The melting endothermic enthalpy and crystallization exothermic enthalpy data are listed in Table 6. The crystallinity of the three PPs could be estimated by their melting endothermic enthalpy. [5] From Table 6, we can see that the melting enthalpy of PP-3 had the highest value of 72.8 J/g, and PP-2 had the lowest value of 51.6 J/g. Due to their different chain stereoregularity, among the three PPs, the crystallinity of PP-3 should be the highest one, and that of PP-2 would be the lowest, which could be verified by XRD analysis with the crystallinity of PP-3 of 62.4% and of PP-2 of 51.1%.

**Figure 6:**
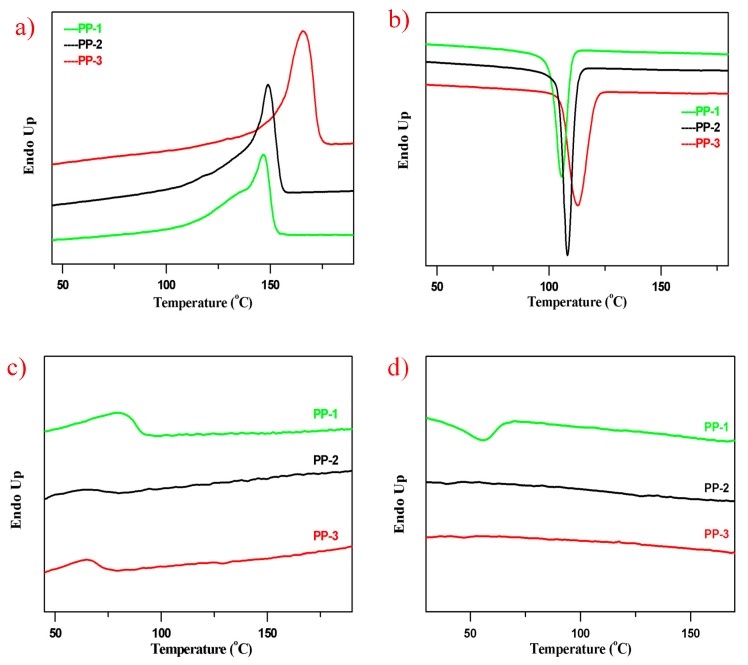
DSC curves of non-soluble and soluble fraction of the PP pellets from xylene at 25 °C (10 °C/min). (**a**) Melting curves of non-soluble fractions of the three samples; (**b**) cooling curves of non-soluble fraction; (**c**) melting curves of soluble fraction; (**d**) cooling curves of soluble fraction. Endo up: the heat flow is endothermic when the peak is up.

The xylene soluble fractions and non-soluble fractions of the three PPs were also characterized by the DSC analysis. As seen from Figure 6, the non-soluble fractions as the major parts of the three PPs had similar melting and cooling curves, and a slightly higher melting peak of the non-soluble fractions than their original PPs could be noticed due to their much higher chain regularity. No obvious crystallization peaks of the xylene soluble fractions in PP-2 and PP-3 were observed, which accounted for the E/P rubber phase generated in the gas phase reactor. The melting peaks of xylene soluble fractions in PP-2 and PP-3 were also lower than in PP-1.

**Figure 7:**
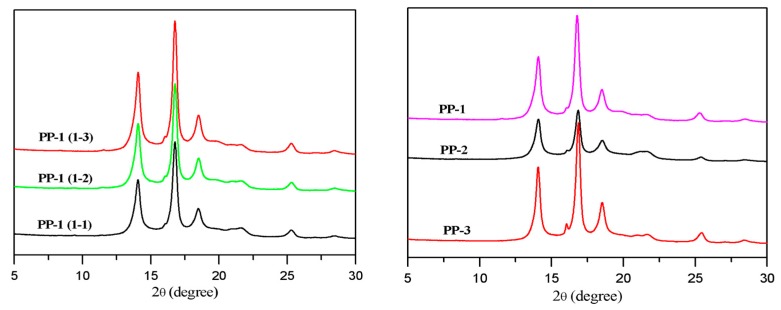
Wide x-ray diffraction results.

Wide angle X-ray diffraction (WXRD) analysis was conducted to characterize the crystal type and content of the prepared PPs. From Figure 7, the diffuse peaks of α crystal of PP could be observed with 2θ of 14.1°, 16.8°, and 18.6°, which were attributed to the (110), (040), and (130) crystal faces, respectively. The crystal types of the PP-1 samples with different NX 8000 content were composed mainly of α crystal. The crystallinity increased from 55.6% to 62.3% with the nucleating agent added in the PP-1 samples from 1500 ppm to 3000 ppm. A tiny characteristic peak of β crystal around 16.1° of the (300) crystal faces could be seen in those samples. The peak area of β crystal slightly increased when the nucleating agent was raised to 3000 ppm. Comparing the different types of PPs with the same additive formula, PP-3 had the highest crystallinity (62.4%) and contained a tiny, but higher β crystal content (about 0.5%), as shown in Figure 7, which might partially explain its higher impact strength than PP-2 with a crystallinity of 51.1% and a trace of β crystal content. The characteristic peak around 20.5° corresponding to the γ crystal was not observed, indicating that the γ crystal in those PPs could be neglected.

### 3.4. TREF Results

**Figure 8:**
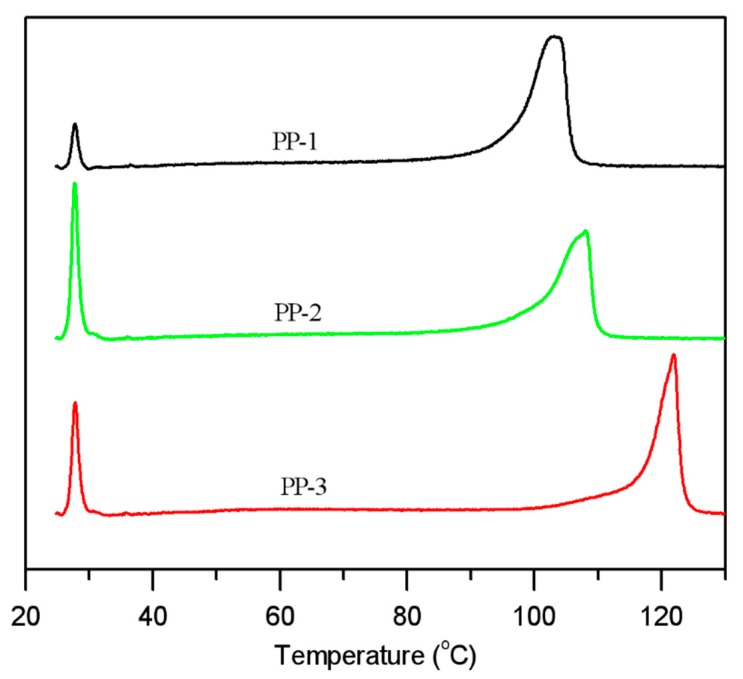
Temperature rising elution fractionation (TREF) results.

The TREF technique was also performed to analyze the chemical composition and chemical composition distributions of PPs. TREF analysis results of the three PPs are presented in Figure 8 and Table 7. The soluble fractions in TREF analysis had similar results as the xylene soluble fractions due to the similar solvent used. The random E/P/B copolymer PP-1 had a 4.0% soluble fraction due to the relatively small noncrystalline molecular chains, and PP-2 had a 17.1% soluble fraction attributed to a small part of the random E/P copolymer with small molecules produced in the loops and a majority of the large rubber molecular chains generated in the gas phase reactor. Similarly, PP-3 had a soluble fraction of 12.1%, which consisted of atactic homo-polypropylene chains from the loops and rubber phase molecular chain from the gas phase reactor.

The TREF curves also could exhibit the comonomer content distributions of copolymers and the chain regularity of homo-polypropylene. As seen from the TREF curves of PP-3, the peak around 121.4 °C with about a 74.6% peak area was typically the highly isotactic polypropylene chains produced in the two loops, and there were also several minor peaks around 89.5 °C, 77.1 °C, and 62.9 °C, which could be ascribed to less isotactic polypropylene chains and E/P copolymer chains. Normally, the crystallization capacity of polypropylene chains increased with lowered ethylene insertion content and increased chain isotacticity. When the ethylene comonomer with a low concentration was added in the two loops, ethylene would insert into the isotactic polypropylene chain to produce the random E/P copolymer with decreased crystallization capacity. As a result, PP-2 had a lower peak around 107 °C than the isotactic polypropylene in PP-3, and the content in this peak was 75.4%, which was close to that of the homopolypropylene produced in the two loops in PP-3. The polymers with high ethylene concentration generated in the gas phase reactor largely formed the rubber phase, thus being soluble in xylene and TCB at room temperature. The major peak in PP-1 was around 103.2 °C with an 89.5% peak area, which was attributed to the insertion of ethylene and 1-butene comonomers in the isotactic polypropylene chain.

Moreover, the different ethylene contents determined by IR in the non-soluble fractions in xylene at 25 °C could be explained reasonably by the TREF curves. There were minor peaks at a lower elution temperature consisting of higher ethylene insertion into the E/P copolymer; therefore, the ethylene content in the non-soluble fraction of PP-2 (4.6–5.8%) could be higher than PP-1 (1.6–1.8%) and PP-3 (1.6–2.0%), despite the fact that the elution temperature of its major peak (107.4 °C) was higher than that of PP-1 (103.2 °C). These major elution peaks in TREF results of the three PPs were in good accordance with the melting peaks in the DSC analysis.

### 3.5. Crystallization and Rubber Phase Morphology

**Figure 9:**
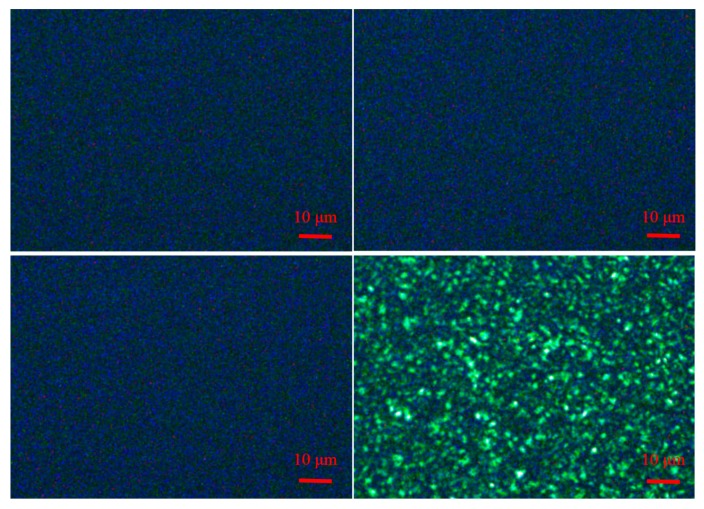
Polarized optical microscope images of PP: (**a**) PP-1, (**b**) PP-2, (**c**) PP-3 with 3000 ppm NX8000, respectively, and (**d**) PP-1 without the nucleating agent as a comparison. (140 °C, 5min).

The isothermal crystallization of the different PPs was characterized by polarized optical microscope (POM). The POM images in Figure 9 clearly showed that the nucleating agent NX8000 had a remarkable influence on the isothermal crystallization of the three PPs by crystallization refinement. We could observe that no crystallization appeared in the three different kinds of PPs (with 200 × magnification) when 3000 ppm NX8000 were added to the three PPs. In contrast, when no nucleating agent was added to PP-1, spherulites (> 1 μm) could be apparently observed.

**Figure 10:**
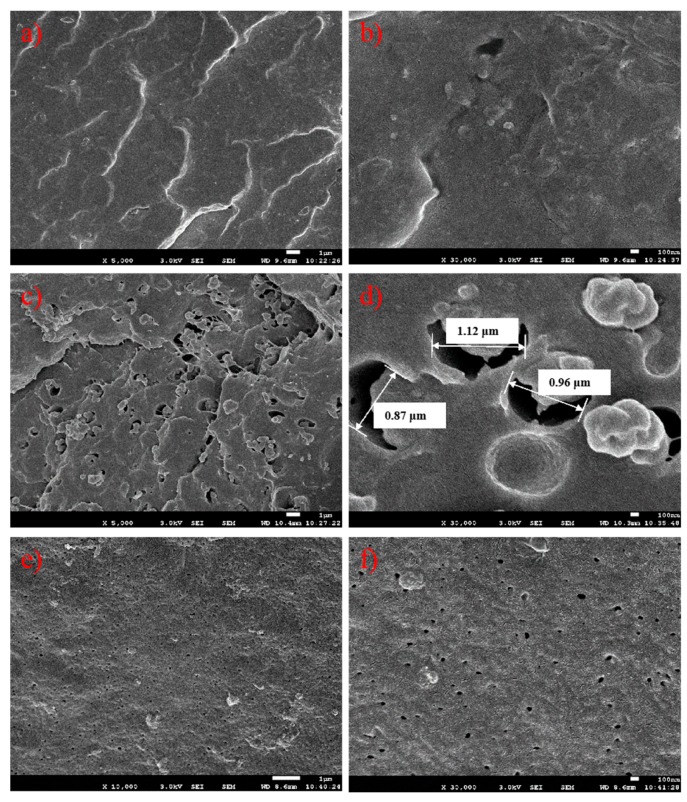
Scanning electron microscopy images of the three PP samples. (**a**) and (**b**) PP-1; (**c**) and (**d**) PP-2; (**e**) and (**f**) PP-3.

The rubber phase size of the three PPs was also characterized by scanning electron microscopy (SEM). The rubber phase was etched by xylene from the cryogenic fraction of injection molded bars. We could see different rubber phase sizes from the three PPs in Figure 10. Apart from a few fractions or rubber phase domains with dimensions of about 200–500 nm, a great deal of tiny rubber holes with diameters of less than 100 nm could be observed on the surface of the E/P/B random copolymer PP-1. In contrast, the impact E/P copolymer PP-2 had many large rubber holes (about 1 μm) on the fraction section. Due to the massive rubber phase dispersed in the PP matrix, PP-2 obtained better impact strength than PP-1. On the other hand, the rubber phase with large domain sizes strongly scattered visible light, thus leading to poor transparency. When the rubber phase size decreased from about 1 μm to about 100 nm, PP-3 obtained excellent impact strength and transparency with an evenly-dispersed rubber phase.

Combining the rubber phase sizes in the SEM images with the GPC analysis of solubility in xylene, we could reasonably infer that the rubber phase with small molecular chains had better thermal compatibility with the semi-crystalline PP matrix, and eventually led to smaller rubber phase sizes after melt blending. When the rubber phase size decreased below the half wavelength of visible light, both PP-1 and PP-3 gained excellent transparency.

## 4. Conclusions

Different types of propylene-based copolymers, including random PP, impact PP, and impact PP with high clarity, could be produced in the Spheripol II process through tuning the comonomer and hydrogen concentration, combining the two loop reactors and the gas phase reactor. The molecular microstructure differences had a significant influence on the mechanical, thermal, and optical properties, as well as the meso-scale rubber phase size. The molecular structure and crystalline and rubber phase size were elucidated by the GPC, ^13^C NMR, TREF, DSC, XRD, POM, and SEM techniques. The results showed that the E/P rubber phase generated in the third gas phase reactor could improve the impact strength dramatically from PP-1 of 6.5 kJ/m^2^ to PP-2 of 14.5 kJ/m^2^. Furthermore, the molecular weight of the E/P rubber phase or xylene soluble fractions at 25 °C could play a key role in the meso-scale dispersion of E/P rubber phase in the PP matrix. The relatively low molecular weight of the rubber phase could promote good thermal compatibility with the semi-crystalline PP chains due to less chain entanglement, resulting in a relatively small rubber phase size. Therefore, the random PP (PP-1) achieved excellent optical transparency with a haze of 12.5%, while the impact PP (PP-2) looked opaque with a haze of 98.8% due to the large rubber size (about 1 μm). The impact PP with high clarity (PP-3) obtained not only good mechanical properties with a flexural modulus of 914 MPa and an impact strength of 25.1 kJ/m^2^, but excellent optical transparency with a haze of only 13.5%, due to its even dispersion and the small dimensions of the rubber size (about 100 nm) in the PP matrix. This new approach to controlling rubber phase size in the PP alloy would be a potentially important strategy for the fabrication of novel high performance PP products.

## Figures and Tables

**Table 1:** Polymerization comonomers used in the reactors of the Spheripol process for the preparation of PP-1/PP-2/PP-3.

Sample	1^st^ Loop	2^nd^ Loop	Gas-Phase Reactor
**PP-1**	propylene/ethylene/1-butene/hydrogen	propylene/ethylene/hydrogen	--
**PP-2**	propylene/ethylene/hydrogen	propylene/ethylene/hydrogen	propylene/ethylene/hydrogen
**PP-3**	propylene/hydrogen	Propylene/hydrogen	propylene/ethylene/hydrogen

**Table 2:** Mechanical analysis results of the PP materials.

Entry	Sample	*Millad NX 8000 ppm	Melting Index g/10 min	Flexural Modulus MPa	Tensile Yield Stress MPa	Impact Strength kJ/m^2^	Heat Distortion Temperature °C	Transmittance %	Haze%	Xc, WAXD %
1	PP-1 (1-0)	0	11.6	891	23.4	3.5	67	88.6	45.1	52.3
2	PP-1 (1-1)	1500	10.9	1005	26.7	4.8	70	88.5	40.6	55.6
3	PP-1 (1-2)	2500	10.6	1139	28.2	6.0	74	87.1	17.8	59.9
4	PP-1 (1-3)	3000	10.6	1162	28.3	6.5	76	87.1	12.5	62.3
5	PP-2	3000	10.8	739	19.6	14.5	69	73.3	98.8	51.1
6	PP-3	3000	8.4	914	25.2	25.1	62	84.2	13.5	62.4

* Other additives: 1010/168/calcium stearate = 500/1000/500 ppm.

**Table 3:** Chemical composition of the pilot pellets and xylene soluble fraction at 25 °C.

Sample	Ethylene Content ^*a^ (wt.%)	1-Butene Content (wt.%)	Xylene Soluble at 25 °C (wt.%)	Ethylene Content of Soluble Fraction in Xylene (wt.%)	Ethylene Content of Non-Soluble Fraction in Xylene (wt.%)
**PP-1**	2.7–3.0	1.1%	4.8	12.0~14.2	1.6~1.8
**PP-2**	8.0	-	15.2	34.7~35.4	4.6~5.8
**PP-3**	4.9	-	12.2	24.6~25.0	1.6~2.0

^*a^ Determined by IR quantitative analysis.

**Table 4:** GPC results.

Sample	Mw ^b^ (g/mol)	Mn (g/mol)	Polydispersion Index (PDI)
PP-1	221,700	38,300	5.79
PP-1-NF ^*b^	175,500	48,500	3.62
PP-1-SF ^*c^	29,700	4500	6.65
PP-2	245,300	36,400	6.73
PP-2-NF	183,600	41,800	4.39
PP-2-SF	292,900	19,000	15.45
PP-3	228,100	42,800	5.33
PP-3-NF	171,900	45,800	3.75
PP-3-SF	95,800	9400	10.18

NF ^*b^: xylene non-soluble fraction at 25 °C, SF ^*c^: xylene soluble fraction at 25 °C.

**Table 5:** The triad and diad distributions of ethylene/propylene copolymers obtained by ^13^C NMR.

Sample	EEE (% ^*d^)	EEP+PEE (%)	PEP (%)	EPE (%)	EPP+PPE (%)	PPP (%)	EE (%)	EP+PE (%)	PP (%)
**PP-1**	1.47	0.37	3.53	0.11	5.92	88.60	1.66	6.78	91.56
**PP-2**	2.94	9.91	4.40	1.47	15.78	65.50	7.90	18.71	73.39
**PP-3**	1.65	4.96	4.13	1.65	9.92	77.69	4.13	13.22	82.65

^*d^: Molar fraction of comonomers in the three PPs determined by NMR. E: ethylene, P: propylene, EEE sequence: triple ethylene molecules in series connection. PP sequence: two propylene molecules in series connection. Similar nomenclature with other sequences.

**Table 6:** DSC analysis results ^*d^.

Sample	Tm (Peak) °C	ΔHm J/g	Tc (Peak) °C	ΔHc J/g
PP-1	144.6	60.6	102.0	−79.8
PP-1-NF	146.6	57.2	105.6	−70.7
PP-1-SF	79.9	8.3	55.8	−5.1
PP-2	148.6	51.6	105.2	−69.0
PP-2-NF	148.8	70	108.2	−85.5
PP-2-SF	-	-	-	-
PP-3	165.2	72.8	125.5	−80.2
PP-3-NF	165.6	75.7	112.8	−90.3
PP-3-SF	66.1	3.3	-	-

^*d^ 20 °C/min for pellets; 10 °C/min for xylene soluble and non-soluble fractions.

**Table 7:** TREF analysis results.

Sample	Item	Soluble Fraction (SF)	Peak 1	Peak 2	Peak 3	Peak 4
PP-1	T/°C		52	57.3	72.4	103.2
	Area/%	4.0	2.8	1.6	2.1	89.5
PP-2	T/°C		57.3	63.9	72.8	107.4
	Area/%	17.1	3.7	1.9	1.8	75.4
PP-3	T/°C		62	77.1	89.5	121.4
	Area/%	12.1	10.9	1.5	0.9	74.6
